# Meta-Analysis on the Effects of Octreotide on Tumor Mass in Acromegaly

**DOI:** 10.1371/journal.pone.0036411

**Published:** 2012-05-04

**Authors:** Andrea Giustina, Gherardo Mazziotti, Valter Torri, Maurizio Spinello, Irene Floriani, Shlomo Melmed

**Affiliations:** 1 Chair of Endocrinology, Department of Medical and Surgical Sciences, University of Brescia, Montichiari Hospital, Brescia, Italy; 2 Department of Medicine, Endocrine Unit, Azienda Ospedaliera “Carlo Poma”, Mantova, Italy; 3 Oncology, Istituto di Ricerche Farmacologiche “Mario Negri”, Milan, Italy; 4 Novartis Farma, Origgio, Varese, Italy; 5 Pituitary Center, Cedars-Sinai Medical Center, Los Angeles, California, United States of America; University of Cordoba, Spain

## Abstract

**Background:**

The long-acting somatostatin analogue octreotide is used either as an adjuvant or primary therapy to lower growth hormone (GH) levels in patients with acromegaly and may also induce pituitary tumor shrinkage.

**Objective:**

We performed a meta-analysis to accurately assess the effect of octreotide on pituitary tumor shrinkage.

**Data Sources:**

A computerized Medline and Embase search was undertaken to identify potentially eligible studies.

**Study Eligibility Criteria:**

Eligibility criteria included treatment with octreotide, availability of numerical metrics on tumor shrinkage and clear definition of a clinically relevant reduction in tumor size. Primary endpoints included the proportion of patients with tumor shrinkage and mean percentage reduction in tumor volume.

**Data Extraction and Analysis:**

The electronic search identified 2202 articles. Of these, 41 studies fulfilling the eligibility criteria were selected for data extraction and analysis. In total, 1685 patients were included, ranging from 6 to 189 patients per trial. For the analysis of the effect of octreotide on pituitary tumor shrinkage a random effect model was used to account for differences in both effect size and sampling error.

**Results:**

Octreotide was shown to induce tumor shrinkage in 53.0% [95% CI: 45.0%–61.0%] of treated patients. In patients treated with the LAR formulation of octreotide, this increased to 66.0%, [95% CI: 57.0%–74.0%). In the nine studies in which tumor shrinkage was quantified, the overall weighted mean percentage reduction in tumor size was 37.4% [95% CI: 22.4%–52.4%], rising to 50.6% [95% CI: 42.7%–58.4%] with octreotide LAR.

**Limitations:**

Most trials examined were open-label and had no control group.

**Conclusions:**

Octreotide LAR induces clinically relevant tumor shrinkage in more than half of patients with acromegaly.

## Introduction

Somatotropin release-inhibiting factor (somatostatin) acts by signaling through specific receptor subtypes to suppress growth hormone (GH) secretion by pituitary somatotroph tumor cells [1]. Long-acting somatostatin analogues act as somatostatin receptor ligands and are widely used for the treatment of acromegaly either as adjuvant or as primary therapy [2]–[4]. When treated with these drugs approximately 50–75% of patients with acromegaly achieve biochemical control, defined as GH <2.5 µg/L and normal age- and sex-adjusted insulin-like growth factor-I (IGF-I) levels [4], [5]. There is growing evidence that somatostatin analogues also induce tumor shrinkage in patients with acromegaly, although the reduction in tumor size observed is not as dramatic and rapid as that seen in patients with prolactinomas treated with dopamine agonists [5]–[9]. The clinical significance of the effect of somatostatin analogues on tumor shrinkage in patients with acromegaly has been further enhanced by the widespread use of long-acting somatostatin analogues as an alternative first-line therapy to surgical tumor resection. In the first-line clinical setting, control of both GH secretory activity and tumor growth are required in order to achieve comprehensive therapeutic efficacy [10]–[15].

Interestingly, the effects of somatostatin analogues on biochemical control and tumor shrinkage may be dissociated and these therapeutic endpoints require separate evaluation [16]. However, in most published studies biochemical control of acromegaly has been considered the main outcome of somatostatin analogue therapy, while information regarding tumor shrinkage effects has been limited due to a number of factors. These factors include the differences in the number of patients studied, and heterogeneity in study design in terms of patient inclusion criteria, length and type of follow-up, imaging techniques and measurements used, type of treatment (i.e. primary or adjuvant therapy) administered and the use of different somatostatin analogues (i.e. lanreotide and octreotide) [7], [10]–[15], [17]–[51].

Long-acting formulations of octreotide and lanreotide are the only two somatostatin analogues currently approved for the treatment of acromegaly. The two somatostatin analogues have modest differences in their affinity for somatostatin receptor subtypes as well as in their respective pharmacokinetic profiles [52], [53]. We recently performed a systematic review of the literature on the effects of lanreotide on pituitary tumor shrinkage; a meta-analysis was not possible due to the paucity of published results for this drug, especially in its Autogel formulation [9]. In contrast, there is an increasing body of literature concerning the effects of octreotide on tumor shrinkage in patients with acromegaly; octreotide was the first somatostatin analogue introduced into clinical practice, and is still widely used for the treatment of acromegaly [7], [8]. Available analyses have been performed several years ago and therefore on limited number of patients particularly concerning the more modern drug formulations [5], [7], [8]. We therefore performed a meta-analysis focused specifically on both subcutaneous and intramuscular octreotide formulations to objectively investigate the tumor shrinkage effects of this drug in acromegaly. The determinants of the action of this drug were also evaluated.

## Methods

To avoid bias the methods for *post hoc* analysis and inclusion criteria were specified in advance and protocol-defined.

### Types of Studies and Endopoints

The searches were designed to select randomized and non-randomized trials, conducted in patients with acromegaly who were treated with octreotide, and which assessed a tumor shrinkage effect. Sole eligibility criteria were the availability of numerical metrics for tumor shrinkage, as well as a clear definition of a clinically relevant reduction in tumor size. Studies with mixed cohorts of patients treated with either octreotide or lanreotide were excluded, unless results relating to each somatostatin analogue type were reported separately.

The primary endpoint was tumor shrinkage evaluated as a categorical (yes/no) variable. The secondary endpoint was the relative reduction in tumor volume/mass from baseline evaluated in studies where these data were reported [19], [33], [34], [37], [38], [40], [42]–[44].

### Search Strategies for Identification of Studies and Data Extraction

A computerized Medline search up to November 2010 was undertaken to identify potentially eligible studies; no language limitation was applied [Table 1]. The same strategy was used to search Embase. Reference lists from trials, narrative reviews, and systematic reviews selected by electronic searching were hand searched to identify additional eligible trials.

**Table 1:** Search strategy used to identify eligible published clinical trials.

Database	Query no.	Search terms
PubMed	1	"Octreotide"[Mesh]
	2	"Acromegaly"[Mesh]
	3	#1 AND #2
	4	#3 Limits: Humans, Publication Date to 2010–11–30
Embase	1	‘octreotide’/exp
	2	‘acromegaly’/exp
	3	#1 AND #2
	4	#3 AND [humans]/lim NOT [30–11–2010]/sd

Identified studies were reviewed by title, abstract and keywords to select potentially eligible studies. Thereafter, full articles were studied to decide which studies met the inclusion criteria. Eligibility assessment was performed independently by two reviewers, a biostatistician and a clinician; if opinions differed, they were resolved by mutual consensus.

Details of study design, patient characteristics, interventions, and outcomes were independently extracted by two authors [G.M and I.F.], using a data extraction form, pilot-tested on four randomly-selected included studies and refined accordingly. Differences in data extraction were solved by a third reviewer, referring back to the original article.

### Statistical Methods

For the primary endpoint, confidence intervals (CI) for estimates of single study endpoints were obtained using exact methods; chi-square distribution was used to test the association between selected factors and response. Specifically planned evaluations included: treatment (octreotide vs octreotide-LAR), proportion of naive patients, duration of therapy (<1 year vs ≥1 year), type of response (linear vs volume); type of lesion (micro- vs macroadenoma); and biochemical response (“safe” GH and normalized IGF-I; treated as ordered variables and tested for trend effect). Given the expected high heterogeneity among studies, a random effect model was used to account for differences in both effect size and sampling error; the between-studies variance was estimated using the DerSimonian and Laird method; the overall effect was estimated using the inverse variance method; the Q statistic was used to assess study heterogeneity and the degree of heterogeneity not explained by sampling error was quantified using the I^2^ index; Assessment of possible publication bias was performed by visual inspection of the funnel plots and by formal analysis using the Egger’s regression test. Statistical analysis was performed using the SAS System, Release 9.2; forest plots were created using the SAS/GRAPH Annotate Facility.

## Results

### Study Selection

**Figure 1:**
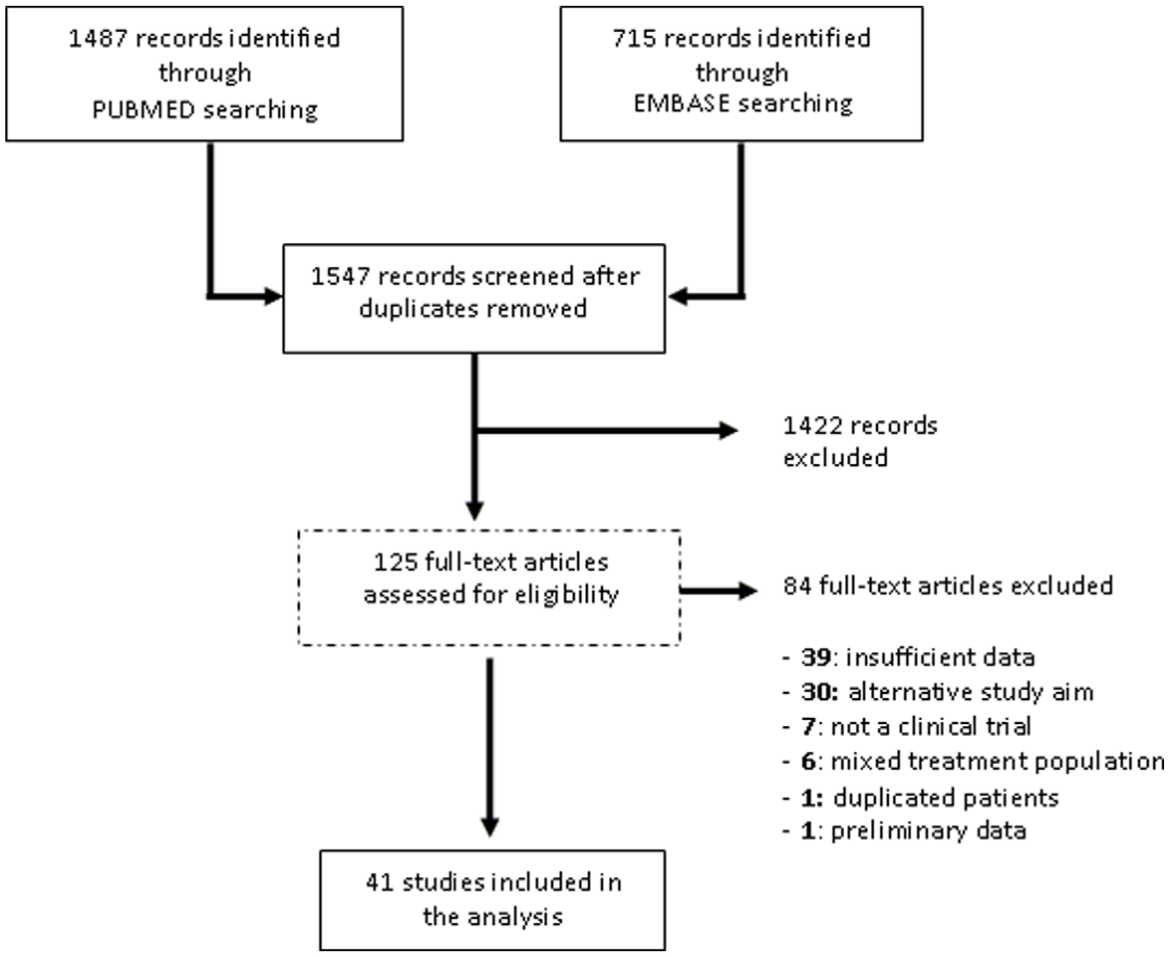
Search strategy and results.

The study selection process is depicted in Figure 1. Electronic searches revealed 2202 articles, of which only 1547 were eligible for the screening. 1422 articles did not meet the eligibility criteria and were discarded. The full text of the remaining 125 studies was fully examined. After examination of the full text, 84 studies were excluded for the following reasons: 39 for reporting insufficient data, 30 because of a different study aim, 7 because they were not clinical trials, 6 because they included a mixed treatment population, 1 because patients were duplicated in another included study, and 1 because the report was preliminary. Accordingly, 41 studies [10]–[15], [17]–[51] fulfilling eligibility criteria were selected for data extraction and analysis.

### Study Characteristics

**Figure 2:**
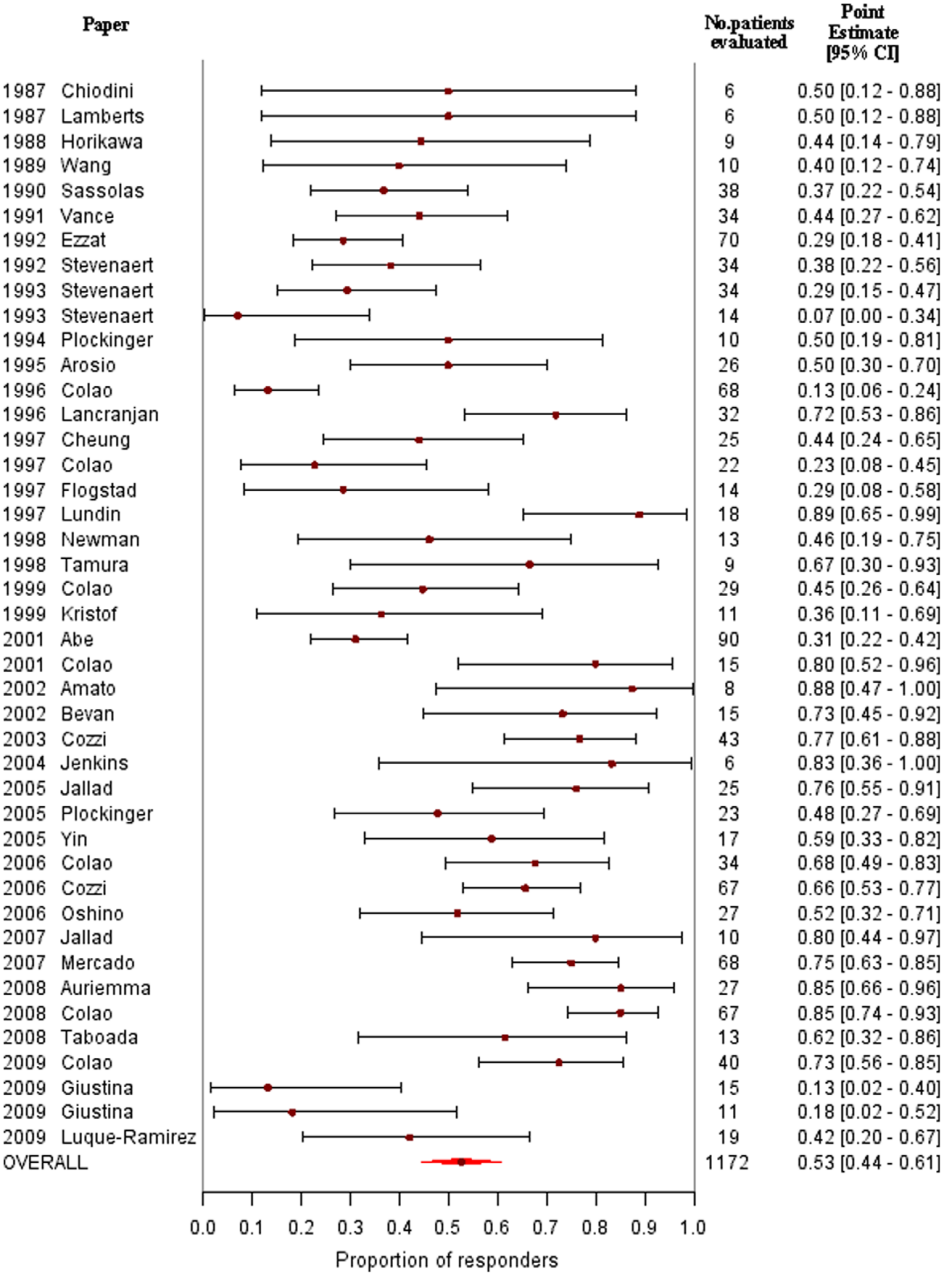
Forest plot depicting the proportion of patients with and without a reduction in tumor size CI, confidence interval.

The characteristics of the 41 eligible studies are shown in Table 2, and the characteristics of the eligible patients and the study results are presented in Table 3. Two studies evaluated two groups of patients with different doses of octreotide [25], [50], and therefore the results of 43 separate studies are presented. Sixteen-hundred-eighty-five patients were included in the evaluable trials, ranging from 6 to 189 patients per trial [Table 2]. Seven-hundred-forty-eight patients were treated with intramuscular octreotide LAR, while the remaining 937 patients received subcutaneous octreotide [Table 2]. Nine hundred and forty-two patients (55.9%) were treated with octreotide as first-line therapy [Table 2]. Data of tumor shrinkage were available for 1172 patients (69.5%), ranging from 6 to 90 per trial [Table 2, Figure 2].

**Table 2:** Characteristics of eligible trials.

Author, year	Design	Enrolled	Analyzed for TS	Treatment naïve pts	Previous			Drug, schedule	Follow–up	TS primary endpoint	Definition of TS
					RTX	SUR	SSA				
Chiodini ‘87	Single, prosp, comp, non–rand	16	6	0%	58.3%	8.3%	0%	OCT sc, 100–300 µg/d	CT, 3 mo	Yes	Reduction >30% of size
Lamberts ’87	Multi, prosp, non–comp	10	6	40%	60%	60%	0%	OCT sc, 200–300 µg/d	CT, 14–16 wk	Yes	Reduction >2 mm max diam
Horikawa ‘88	Single, prosp, non–comp	10	9	60%	10%	20%	0%	OCT sc, 150–800 µg/d	CT/MRI 3–35 wk	Yes	Reduction >20% in volume
Wang ‘89	Single, prosp, non–comp, rand	10	10	40%	30%	10%	0%	OCT sc, different schedules	CT, 6 mo	No	Reduction >25% in volume
Sassolas ‘90	Multi, prosp, non–comp	58	38	32.8%	67.2%	67.2%	0%	OCT sc, 300–1500 µg/d	CT, 6 mo	No	Reduction >20% of size
Vance ’91	Multi, prosp, non–comp	189	34	15.6%	61.5%	26.0%	0%	OCT sc, 100–1500 µg/d	MRI/CT, unk	No	Reduction >20% of size
Ezzat ‘92	Multi, prosp, comp, rand, double–blind	116	70	0%	39%	64%	50%	OCT sc, 300 µg/d	MRI/CT, 6 mo	Yes	Reduction >1 mm max diam
Stevenaert ‘92	Single, prosp, comp, non rand	37	34	70.3%	10.8%	5.4%	No	OCT sc, 300–1500 µg/d	MRI/CT, various	Yes	Reduction >20% of size
Stevenaert‘93	Single, prosp, non–comp	34	34	0%	0%	100%	100%	OCT sc, 300–1500 µg/d	MRI/CT, 2–14 mos	No	Reduction >25% max diam
Stevenaert ‘93bis	Single, prosp, non–comp	14	14	100%	0%	0%	0%	OCT sc, 300 µg/d	MRI/CT, 3 wk	No	Reduction >25% max diam
Plockinger ‘94	Single, prosp, non–comp	10	10	70%	20%	30%	0%	OCT sc, 1500 µg/d	MRI, 3 mo	Yes	Reduction >25% of volume
Arosio ‘95	Multi, prosp, non–comp	68	26	29%	0%	14.7%	0%	OCT sc, 300 µg/d	MRI/CT, 12 mo	No	Reduction >10% of volume
Colao ‘96	Single, retro, non–comp	68	68	64.7%	0%	35.3%	0%	OCT sc 150–600 µg/d	MRI/CT, 3 mo	Yes	Reduction ≥30% of volume
Lancranjan ’96	Multi, prosp, comp, rand	101	32	0%	unk	unk	100%	OCT LAR im, 20–40 mg/28 d	unk	Yes	Reduction >20% of size
Cheung ‘97	Single, prosp, non–comp	27	27	22.2%	55.6%	63.0%	0%	OCT sc, 150–450 µg/d	MRI/CT, various	Yes	Reduction of at least 3 mm
Colao ‘97	Single, prosp, comp, rand	22	22	100%	0%	0%	0%	OCT sc, 150–600 µg/d	MRI/CT, 3–6 mo	Yes	Reduction >30% max diam
Flogstad ‘97	Single, prosp, non–comp	14	14	40%	0%	unk	0%	OCT LAR im, 20–40 mg/28 d	MRI/CT 12 mo	yes	Reduction >20% of size
Lundin ‘97	Single, prosp, non–comp	18	18	94.4%	5.6%	5.6%	0%	OCT sc, 200–2500 µg/d	MRI/CT, various	Yes	Reduction ≥18% volume
Newman ‘98	Multi, prosp, comp, rand	26	13	100%	0%	0%	0%	OCT sc, 300–750 µg/d	MRI, 6 mo	No	Reduction ≥10% max diam
Tamura ‘98	Single, prosp, non–comp	9	9	100%	0%	0%	0%	OCT, 120–240 µg/d	MRI, 4 wk	Yes	Reduction >20% of size
Colao ‘99	Single, prosp, non–comp	38	29	81.6%	0%	18.4%	0%	OCT sc, 150–900 µg/d	MRI/CT, 12 mo	Yes	Reduction >30% of volume
Kristof ‘99	Single, prosp, non–comp	11	11	100%	0%	0%	0%	OCT, 150–900 µg/d	MRI, 3 mo	Yes	Reduction >2 mm in ≥1 plane
Abe ‘01	Multi, prosp, non–comp	90	90	100%	0%	0%	0%	OCT sc, various dose	MRI, various	Yes	Reduction >2 mm max diam
Colao ‘01	Single, prosp, non–comp	36	15	100%	0%	0%	0%	OCT LAR im, 20–40 mg/28 d	MRI, 24 mo	No	Reduction >25% of volume
Amato ‘02	Multi, prosp, comp, rand	8	8	100%	0%	0%	0%	OCT LAR im, 20–30 mg/28 d	MRI 12 mo	Yes	Reduction >10% of volume
Bevan ‘02	Multi, prosp, non–comp	15	15	0%	0%	0%	100%	OCT LAR im, 20–30 mg/28 d	MRI/CT 24 wk	Yes	Reduction >20% of volume
Cozzi ‘03	Multi, retro, non–comp	110	43	23.5%	0%	0%	76.5%	OCT LAR im, 10–30 mg/28 d	MRI, 30 mo	Yes	Reduction >25% of volume
Jenkins ‘04	Single, prosp, non–comp	6	6	100%	0%	0%	0%	OCT LAR im, 20–30 mg/28 d	MRI/CT, 6 mo	Yes	Reduction >20% of volume
Jallad ‘05	Single, prosp, non–comp	28	25	50%	0%	0%	50%	OCT LAR im, 10–30 mg/28 d	MRI, 6 mo	No	Reduction >25% of volume
Plockinger ‘05	Single, prosp, non–comp	24	23	100%	0%	0%	0%	OCT sc, 300 µg/d	MRI, 3 mo	No	Reduction >20%of volume
Yin ‘05	Single, retro, non–comp	17	17	100%	0%	0%	0%	OCT LAR im, 30 mg/28 d	MRI, 3 mo	No	Reduction ≥10% of volume
Colao ‘06	Multi, prosp, non–comp	34	34	100%	0%	0%	0%	OCT LAR im, 20–30 mg/28 d	MRI, 6 mo	Yes	Reduction >30% of volume
Cozzi ‘06	Multi, prosp, non–comp	67	67	100%	0%	0%	0%	OCT LAR im, up to 30 mg/28 d	MRI, 6 mo	Yes	Reduction >25% of volume
Oshino ‘06	Single, prosp, non–comp	32	27	100%	100%	6.2%	100%	OCT sc, 300 µg/d	MRI, 2–3 wk	No	Reduction >2 mm max diam
Jallad ‘07	Single, prosp, non–comp	11	10	100%	0%	0%	0%	OCT LAR im, 20–30mg/28d	MRI, 6 mo	Yes	Reduction >25% of volume
Mercado ‘07	Multi, prosp, non–comp	98	68	100%	0%	0%	0%	OCT LAR im, 20–30mg/28d	MRI, 6 mo	Yes	Reduction >20% of volume
Auriemma ‘08	Single,retro, comp,non rand	27	27	100%	0%	0%	0%	OCT LAR im, 10–30 mg/28 d	MRI, 12 mo	Yes	Reduction >25% of volume
Colao ‘08	Single, retro, obs	67	67	100%	unk	0%	0%	OCT LAR im, 20–30 mg/28 d	MRI, 12 mo	Yes	Reduction >25% of volume
Taboada ‘08	Multi, prosp, non–comp	22	13	100%	unk	100%	unk	OCT LAR im, 20–30 mg/28 d	MRI, 6 mo	Yes	Reduction >25% of volume
Colao ‘09	Multi, prosp, comp, rand, open	40	40	100%	0%	0%	0%	OCT LAR im, 20–30 mg/28 d	MRI, 48 wk	No	Reduction >20% of volume
Giustina ‘09	Multi, prosp, comp, rand, open	16	15	0%	0%	77.8%	100%	OCT LAR im, 60 mg/28 d	MRI 6 mos	No	Reduction >20% of volume
Giustina ’09bis	Multi, prosp, comp, rand, open	12	11	0%	0%	50%	100%	OCT LAR im, 30 mg/28 d	MRI 6 mo	No	Reduction >20% of volume
Luque–Ramirez ‘09	Multi, prosp, non–comp	19	19	100%	0%	0%	0%	OCT LAR im, 20–30 mg/28 d	MRI, 12 mo	Yes	Reduction >25% of volume

CT, computed tomography; comp, comparative; d, day; diam, diameter; max, maximum; mm, millimeter; mo, month; multi, multicenter; MRI, magnetic resonance imaging; non–comp, non-comparative; non–rand, non-randomized; obs, observational; OCT LAR, intramuscular octreotide long-acting repeatable; OCT sc, octreotide subcutaneous; open, open-label; pts: patients; prosp, prospective; rand, randomized; retro, retrospective; RTX, radiotherapy; SSA, somatostatin analogs; single, single-center; SUR, surgery; TS, tumor shrinkage; unk, unknown; wk week.

**Table 3:** Characteristics of included patients, and study results.

Author, year	Micro/Macro adenoma	M/F	Age in yearsMean ± SD (range)	RESPONDER	RESPONSE BY MICRO/MACRO	BIOCHEMICAL RESPONSE	CORRELATION
Chiodini ‘87	unk	1/5	50.2±9.83 (37–64)	3/6	unk	4/12 safe GH	2/3 with TS had safe GH;0/3 without TS had safe GH
Lamberts ’87	unk	7/3^§^	47.3±11.46 (35–66)	3/6	unk	unk	unk
Horikawa ‘88	unk	7/3^§^	40.0±9.3 (25–53)§	4/9	unk	4/9 safe GH, 4/9 IGF-normal, 2/9 both	TS: 2/4 pts safe GH, 2/4 IGF-I normal, 1/4 both;No TS: 2/5 had safe GH, 2/5 safe IGF-I, 1/9 both
Wang ‘89	5/5	5/5	41.1±8.82 (32–58)	4/10	1/5 micro; 3/5 macro	5/10 safe GH	unk
Sassolas ‘90	0/38	28/30^§^	48 (22–74)* ^§^	14/38	unk	12/54 GH normal	unk
Vance ‘91	unk	82/107^§^	49^#^ (18–77)^§^	15/34	unk	82/189 safe GH, 46/99 IGF-I normal	unk
Ezzat ‘92	unk	61/55	46.0*§	20/70	unk	50/98 safe GH, 59/98 IGF-I normal	TS associated with GH and IGF-I reduction
Stevenaert ‘92	unk	22/15^§^	(23.5–64.5)°^§^	13/34	unk	25/37 safe GH and 19/37 IGF-I normal	5/5 pts with TS had safe GH
Stevenaert ‘93	unk	24/24^§^	(23–65)°	10/34	unk	13/34 GH normal, 21/34 safe IGF-I	unk
Stevenaert ‘93bis	unk	24/24^§^	(23–65)°	1/14	unk	3/14 GH normal, 7/14 safe IGF-I	unk
Plockinger ‘94	0/10	4/6	42.3±12.84 (26–67)	5/10	unk	8/10 safe GH, 5/10 IGF-I normal	unk
Arosio ‘95	9/17	25/48^§^	45.9±12 (19–70)^§^	13/26	8/9 micro; 5/17 macro	30.8% safe GH	TS: 38.4% safe GH; No TS: 23% safe GH
Colao ‘96	10/58	33/41^§^	(16–70)°^§^	9/68	unk	28/68 safe GH	7 pts who responded and 2 who did not had TS
Lancranjan ‘96	unk	unk	unk	23/32	unk	95/101 safe GH	unk
Colao ‘97	unk	27/32^§^	18–66^§^°	5/22	unk	13/22 GH normal, 12/22 safe IGF-I GH normal	unk
Cheung ‘97	12/15	13/14	47.2±14.02 (20–72)	11/25	unk	20/27 safe IGF-I	unk
Flogstad ‘97	unk	8/6	52^#^ (27–69)	4/14	unk	13/14 safe GH, 9/14 safe IGF-I	unk
Lundin ‘97	4/14	6/12	51*(31–70)	16/18	unk	12/18 safe GH, 5/18 safe GH IGF-I normal	unk, but shrinkage did not correlate with GH
Newman ’98		10/16^§^	50*(20–78)§	6/13	unk	4/13 safe GH,9/13 IGF-1 normal, 4/13 both	TS: 4/6 IGF-I normal, 1/6 safe GH; No TS: 5/7 IGF-I normal,3/7 safe GH
Tamura ‘98	3/6	5/4	44.5±9.09 (32–55)	6/9	unk	6/9 safe GH	TS: 5/6 safe GH; No TS: 1/3 safe GH
Colao ‘99	unk	15/14	15–68***	13/29	unk	20/29 safe GH and IGF-1 normal,	unk, but significant correlation
Kristof ‘99	1/8	7/4	44.8±15.7	4/11	1/1 micro; 2/8 macro	unk	unk, but no significant correlation
Abe ‘01	7/83	45/45	46.1±1.4** (7–82)	28/90	1/7 micro; 27/83 macro	43/90 safe GH, 33/90 IGF-I normal, 27/90 both	unk
Colao ‘01	3/12	15/21	50.5±14.62 (24–77)	12/15	2/3 micro 10/12 macro	12/15 pts with safe GH, 8/15 with IGF-I normal	9/15 GH and TS; 7/15 IGF-I and TS; 6/15 GH, IGF-I and TS
Amato ‘02	4/4	3/5	52.2±11.46 (40–68)	8/8	unk	3/8 safe GH,3/8 IGF-I normal	unk
Bevan ‘02	5/10	17/10^§^	53*(21–73)^§^	11/15	unk	11/14 safe GH, 8/24 IGF-I normal, 7/24 both	4/14 had safe GH, IGF-I normal and TS
Cozzi ‘03	15/61§	48/62^§^	55^#^ (21–85)^§^	33/43	unk	37/51 safe GH, 34/51 IGF-I	unk
Jenkins ‘04	2/4	unk	53 (42–76)	5/6	unk	3/6 safe GH; 2/6 IGH normal	No correlation between TS and serum GH and IGF-I
Jallad ‘05	3/23	34/46^§^	43.0±12.9 (18–80)^§^	19/25	unk	20/27 safe GH, 11/27 IGF-I normal	TS: 12/19 IGF-I normal, with significant correlation
Plockinger ‘05	0/24	11/13	45* (29–70)	11/23	all macro	14/24 safe GH, 20/24 IGF-I normal	unk
Yin ‘05	6/11	10/7	41±9.6* (23–56)	10/17	1/6 micro; 9/11 macro	6/17 safe GH	unk
Colao ‘06	13/21	20/14	50.0±9.6 (31–64)	23/34	7/13 micro; 16/20 macro	19/34 safe GH, 15/34 IGF-I	unk
Cozzi ‘06	19/48	31/36	54.9±14.2	44/67	10/19 micro; 39/48 macro	68% safe GH, 70.1% IGF-I normal, 56.7% both	44.8% had TS+biochemical control; 35% only TS; 3% only biochemical control
Oshino ‘06	3/24§	18/14^§^	45.6* (22–68)^§^	14/27	1/3 micro; 13/24 macro	6/32 safe GH, 4/32 IGF-I normal	Uncorrelated, but not specified
Jallad ‘07	1/10	8/3	42.4±11.7 (25–70)	8/10	unk micro; 8/10 macro	4/11 safe GH, 9/11 IGF-I normalization	2/8 pts with TS did not reach IGF-I normalization
Mercado ‘07	8/60	28/40	49.7±13.2 (24–77)	51/68	8/8 micro; 43/60 macro	29/60 safe GH,26/60 IGF-I normal,17/68 both	Uncorrelated, but not specified
Auriemma ‘08	unk	16/11	48.4±17.4	23/27	unk	21/27 safe GH, 17/27 IGF-I normal, 17/27 both	unk
Colao ‘08	22/45	34/33	44.4*	57/67	20/22 micro; 37/45 macro	35/67 GH control and 39/67 IGF-I normal	TS significantly correlated with GH and IGF-I levels
Taboada ‘08	unk	11/11	40^#^ (24–62)	8/13	unk	9/22 safe GH and IGF-I normal	unk
Colao ‘09	unk	24/16	45±12.5 (20–76)	29/40	unk	11/40 safe GH and IGF-I normal at 48 wk	unk
Giustina ’09	unk	6/10	50^#^ (32–78)	2/15	unk	0/15 safe GH; 0/15 IGF-I normal	unk
Giustina ’09bis	unk	8/4	51^#^ (27–79)	2/11	unk	3/11 safe GH; 4/11 IGF-I normal; 2/11 both	unk
Luque–Ramirez ‘09	5/14	8/11	52±14 (29–79)	8/19	3/5 micro; 5/14 macro	7/13 safe GH, 6/13 IGF-I normal	unk

* Mean;

** Mean+SE; #Median; ^§^description of the total sample; °min–max;

*** min–max;

GH, growth hormone; IGF-I, insulin-like growth factor-I; M/F, male/female; macro, macroadenoma; micro, microadenoma; TS, tumor shrinkage; unk, unknown.

Of the 41 studies, 32 measured tumor response according to adenoma volume or size (cut-off varying from 10% to 30%), whereas in the remaining 9 studies tumor shrinkage was defined according to the decrease in the largest measurable adenoma diameter [Table 2]. In over 50% of the studies, the pituitary tumor was evaluated by magnetic resonance imaging whereas computerized tomography was used in 18 studies (Table 2). The duration of therapy ranged from 2 weeks to 30 months [Table 2]. Twelve studies described a tumor shrinkage effect according to the type of lesion (micro/macroadenoma), 37 provided information on the percentage of patients with “safe” GH levels: i.e., random GH levels below 2.0–2.5 µg/L in 23 studies [11]–[15], [20], [21], [25], [27], [36]–[42], [44], [46]–[51], random GH below 5 µg/L in 12 studies [17]–[19], [22], [23], [28], [29], [31]–[34], [43] and suppressed GH values during oral glucose tolerance test in two studies [30], [35]. Thirty-one studies reported the percentage of patients with normal IGF-I levels [Table 3].

### Tumor Shrinkage

#### Meta-analysis

**Figure 3:**
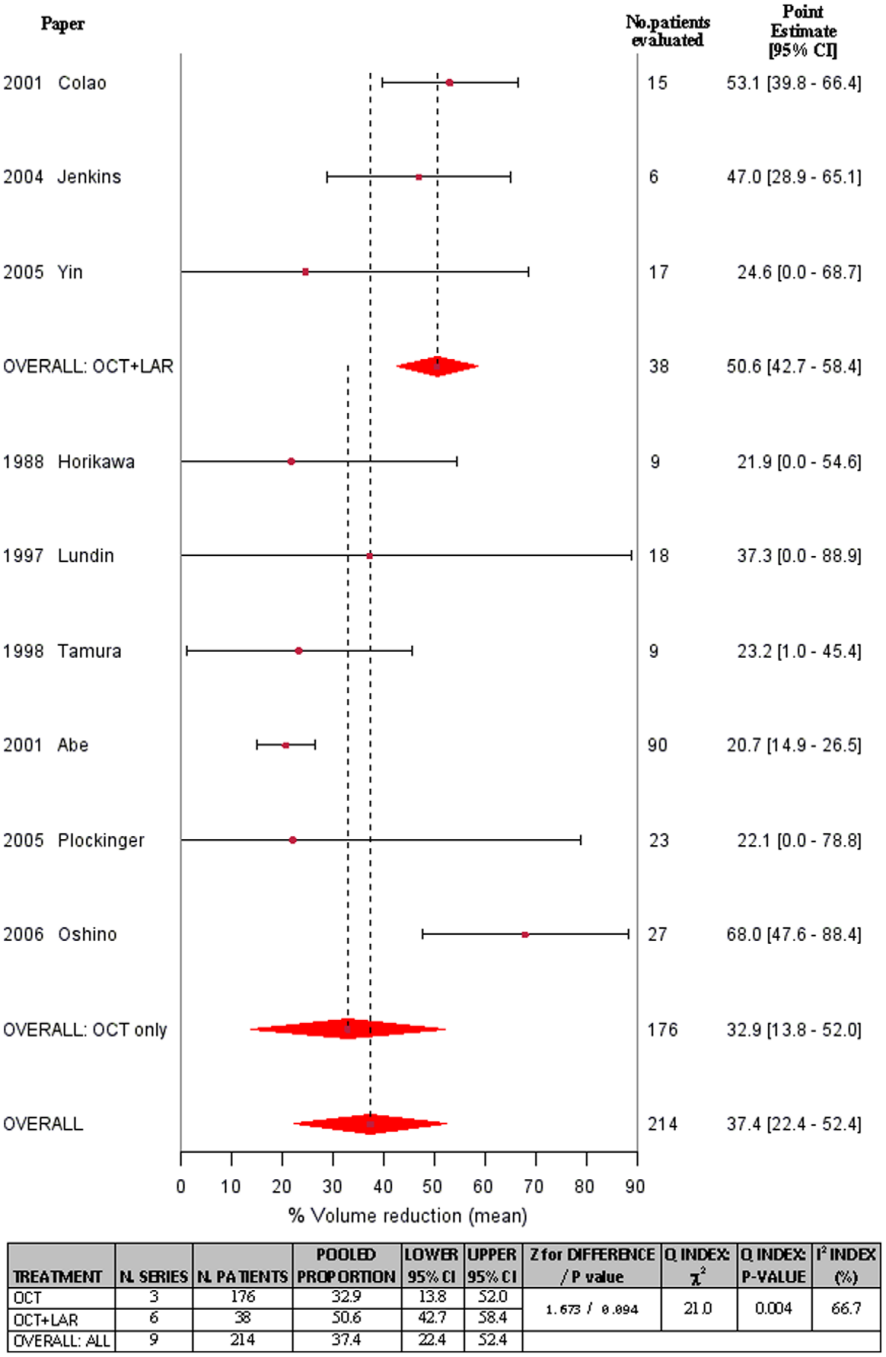
Forest plot depicting percentage change in tumor volume CI, confidence interval.

Overall, in the 43 groups of patients evaluated in the 41 studies, 53.0% (95%CI: 45.0%–61.0%) of patients demonstrated a reduction in tumor size (Figure 2). Heterogeneity in tumor reduction was very high (χ^2^ for heterogeneity: 433.850, p<0.001; I^2^ = 90.3), and was not explained by the use of a different measurement of shrinkage (linear vs volumetric): I^2^ reduced to 87.5 from 90.3 when stratifying by type of measurement, and was still very high in both subgroups (linear: I^2^ = 58.1; volumetric: I^2^ = 89.6). No evidence for a possible publication bias was detected (p-value for the Egger’s test: 0.694). When the analysis was restricted to studies in which stringent criteria for tumor reduction were used (i.e., volume decrease of at least 20%) [Table 2], 57.0% (95% CI: 47.0%–67.0%) of patients exhibited tumor shrinkage. When the analysis was restricted to studies in which tumor shrinkage was assessed by MRI, 60.0% (95% CI: 51.0%–70.0%) of patients showed tumor shrinkage [Figure S1]. Moreover, when the analysis was restricted to studies with follow-up longer than 3 months, tumor shrinkage was seen to occur in 59.0% of patients (95% CI: 50.0%–68.0%) [Figure S2]. In an analysis of the 9 studies in which the degree of shrinkage was reported, the weighted mean percentage reduction in tumor size was 37.4% (95% CI: 22.4%–52.4%), with the greatest effects observed in patients treated with octreotide LAR compared with subcutaneous octreotide (50.6% [95% CI: 42.7%–58.4%] vs 32.9% [95% CI 13.8%–52.0%], respectively; p = 0.094) [Figure 3].

**Table 4:** Univariate analysis of factors associated with tumor response.

CATEGORY	NO IN SERIES	NO PATIENTS	POOLED PROPORTION (95% CI)	POOLED ODDS RATIO (95% CI)	P–VALUE	Q INDEX: χ^2^	Q INDEX: P–VALUE	I^2^ INDEX (%)
**TYPE OF MEASUREMENT**								
LINEAR	10	312	0.32 (0.23–0.40)	Ref	<0.001	331.544	<0.001	87.6
VOLUME/SIZE	33	860	0.58 (0.49–0.68)	2.73 (1.73–4.31)				
**SIZE OF TUMOR MASS**								
MICRO	12	451	0.58 (0.46–0.71)	Ref	0.962	182.012	<0.001	86.8
MACRO	14	484	0.59 (0.47–0.70)	1.02 (0.51–2.01)				
**PROPORTION OF PATIENTS WITH ‘SAFE’ GH LEVELS**								
0– ≤25%	4	94	0.27 (0.07–0.47)	Ref	<0.001(test for trend)	297.480	<0.001	88.9
25– ≤50%	15	397	0.50 (0.36–0.64)	2.26(0.76–6.73)				
50– ≤75%	12	393	0.57 (0.43–0.72)	3.37 (1.11–10.30)				
75– ≤100%	6	113	0.67 (0.51–0.83)	5.00 (1.50–16.63)				
**PROPORTION OF PATIENTS WITH NORMALIZED IGF-1**								
0– ≤25%	2	42	0.32 (0–0.70)	Ref	0.039(test for trend)	297.375	<0.001	90.9
25– ≤50%	14	359	0.55 (0.40–0.70)	2.78 (0.39–20.11)				
50– ≤75%	13	408	0.53 (0.38–0.68)	2.53 (0.35–18.46)				
75– ≤100%	2	33	0.63 (0.32–0.95)	3.62 (0.34–38.42)				
**% TREATMENT NAIVE PATIENTS**								
0%	7	183	0.40 (0.22–0.58)	ref	<0.001(test for trend)	349.753	<0.001	88.6
0– ≤75%	13	342	0.45 (0.31–0.60)	1.23 (0.48–3.14)				
75– ≤100%	23	647	0.60 (0.50–0.71)	2.36 (0.96–5.78)				
**TREATMENT DURATION**								
<1 YEAR	26	683	0.46 (0.36–0.56)	Ref	0.043	291.927	<0.001	87.0
≥1 YEAR	14	414	0.61 (0.50–0.73)	1.90 (1.02–3.55)				
**TREATMENT**								
OCT	23	626	0.41 (0.32–0.49)	Ref	<0.001	224.462	<0.001	81.7
OCT-LAR	20	546	0.66 (0.57–0.74)	3.18 (1.95–5.20)				

CI, confidence interval; OCT, octreotide; OCT-LAR, octreotide long-acting repeatable; MICRO, microadenoma; MACRO, macroadenoma; GH, growth hormone; IGF-I, insulin-like growth factor-I.

#### Analysis of determinants of tumor shrinkage

The effect of treatment on tumor shrinkage was affected by several different factors [Table 4]. Treatment resulted in significantly greater tumor shrinkage if 1) tumor dimensions were reported as volume rather than as a linear measurement (odds ratio [OR]: 2.73; 95% CI: 1.73–4.31; p<0.001); 2) patients had been treated with octreotide LAR rather than subcutaneous octreotide (OR: 3.18; 95% CI: 1.95–5.20); p<0.001); 3) patients had a treatment duration longer than 1 year as compared with a shorter treatment duration (OR: 1.90; 95% CI: 1.02–3.55; p = 0.043); or 4) patients had received octreotide as first-line therapy (OR increased according to the higher proportion of treatment-naïve patients: p<0.001).

The correlation between tumor shrinkage and biochemical response was also assessed; a positive relationship between tumor shrinkage and the achievement of “safe” GH levels was observed: in studies where higher rates (>75%) of “safe” GH levels were observed, more patients experienced tumor shrinkage compared with those studies in which “safe” GH control was less frequent (<25%) (OR: 5.0; 95% CI 1.50–16.63; Table 4); a positive correlation between tumor shrinkage and the attainment of normal IGF-I level was also observed [Table 4]. Moreover, tumor shrinkage did not correlate with the initial pre-treatment tumor size [Table 4]. The unexplained variability, however, was always >80% even after considering the effect of each of these variables.

## Discussion

This meta-analysis shows that, overall, clinically significant tumor shrinkage occurs in more than 50% of patients with acromegaly treated with octreotide. Octreotide was the first somatostatin analogue used for the treatment of acromegaly in 1984 and subsequently thousands of patients with acromegaly have been treated with this agent [54], [55]. The rationale for using octreotide, like other somatostatin analogues, in the treatment of acromegaly is based on the well known effect of somatostatin in suppressing GH hypersecretion by pituitary tumor cells [1], [56]. Besides biochemical effects, somatostatin and its analogues also inhibit tumor cell growth [7], [8]. This effect is of particular clinical relevance because somatostatin analogues are increasingly being used as first-line therapy in patients with acromegaly [10], [59].

To date, several analyses have been published on the effects of somatostatin analogues on tumor shrinkage in patients with acromegaly [5], [7]–[9]. Results of these critical analyses clearly established that tumor shrinkage may occur in 40–50% of acromegaly patients treated with somatostatin analogues, particularly when these drugs were used as first-line therapy [7], [8]. Indeed, it has been suggested that somatostatin analogues may control tumor growth in nearly all patients, since very few patients experienced persistent tumor enlargement during medical therapy [7]. However, at the time these analyses were published, available data were sparse, particularly for octreotide LAR, which has only been introduced into clinical practice in the past decade [60].

In recent years, a wealth of new clinical studies examining the effects of somatostatin analogues including octreotide LAR in patients with acromegaly has been published [9], [13]-[15], [41]–[51]. However, comparison of these studies in terms of their effects on tumor shrinkage is challenging since they differ greatly in their design. For example, there is marked heterogeneity in the length and type of patient follow-up, the use of imaging techniques and tumor measurements, the type of treatment administered (i.e. primary or adjuvant therapy) and the type of drug employed [9], [13]–[15], [41]–[51]. Consequently, we performed this meta-analysis to objectively assess the magnitude of tumor shrinkage using all the available data. We focused on octreotide, since data on lanreotide Autogel (the other somatostatin analogue currently available in clinical practice) are still too sparse to allow a rigorous meta-analysis to be undertaken [9].

The studies identified by this meta-analysis have highly heterogeneous study designs, and employed different criteria to define tumor mass before and after therapy. Some studies employed absolute or percentage changes in tumor diameter, whereas others used absolute or percentage changes in tumor volume. Our meta-analysis showed that the percentage of patients experiencing tumor shrinkage was higher in studies reporting changes in tumor volume than in those measuring changes in tumor diameter. This observation is consistent with the assumption that three-dimensional tumor measurements are more reliable in detecting even small changes in tumor size [61].

Inherent limitations of all studies dealing with tumor shrinkage are the arbitrarily chosen criteria used to define the clinical significance of the treatment effect. This limitation is amplified when a meta-analysis like this is performed, due to the reliance on a single center definition of tumor shrinkage. Moreover, the applied criteria were based exclusively on radiological evaluation; clinical endpoints (e.g., improvement of visual fields) were not consistently considered in single publications. Nevertheless, it is now accepted that a 20% decrease in tumor size (volume or diameter) may be considered a significant shrinkage. This metric is reflective of the technical variability of assessment methods (which are not believed to exceed this figure), the average baseline adenoma dimensions (about 1.5–2.0 cm) in acromegaly and the potential beneficial effects of relieving compression of surrounding structures. Interestingly, several studies have reported longitudinal data for the magnitude of tumor shrinkage [19], [33], [34], [37], [38], [40], [42]–[44]. Therefore, our meta-analysis defined the phenomenon in terms of prevalence and provided a quantitative evaluation. Notably, while for short-acting subcutaneous octreotide, the average shrinkage effect was just slightly higher than the threshold of clinical significance, the mean reduction in tumor size in patients treated with octreotide LAR was almost 50%. This strongly suggests that the effects of octreotide LAR are more than a simple radiological phenomenon and have significant implications for clinical practice.

Pituitary adenoma shrinkage is an important clinical effect of somatostatin analogues particularly when used as first-line treatment of acromegaly. Primary somatostatin analogue therapy may be offered in selected patients with unacceptable anesthesiological risk and in those harboring macroadenomas with little likelihood of surgical cure [62]. In these situations the tumor shrinkage effect of somatostatin analogues is desirable in addition to biochemical disease control. This meta-analysis demonstrated that octreotide, when used as first-line therapy, may produce tumor shrinkage in about two-thirds of patients with acromegaly, whereas this effect was seen less frequently when the drug was used after surgical resection and/or radiotherapy. This finding may also be explained, at least in part, by difficulties in evaluating tumor shrinkage in patients who have previously undergone surgery or radiotherapy. In fact, transsphenoidal resection induces anatomical pituitary and sellar alterations that result in poor reproducibility when evaluating pituitary imaging [63]. Surgical packing materials placed into the sella may re-absorb and the volume of the residual mass may decrease, mimicking a shrinkage effect. Previous radiotherapy may also alter the results of pituitary imaging by causing fibrotic changes in the sellar content, which prohibit precise tracing of tumor margins [63].

This meta-analysis also demonstrated that intramuscular octreotide LAR produced tumor shrinkage in twice as many patients as subcutaneous octreotide. A similar size effect was observed with the quantitative analysis when the magnitude of tumor shrinkage was evaluated. It is unlikely that such a difference is attributable to technical reasons, such as differences in the resolution of radiological tools employed in the older studies, in which subcutaneous octreotide was evaluated, and the more recent studies in which octreotide LAR was assessed [7]. It is more likely that the advantage of octreotide LAR as compared to subcutaneous octreotide, observed in our meta-analysis, reflects a true difference between the two formulations. Similar findings were observed with lanreotide, when the Autogel formulation was compared to the shorter term SR formulation [9]. This provides convincing evidence that the biological effects of somatostatin analogues may be influenced by their pharmacokinetic profiles; prolonged and constant exposure of tumor cells to somatostatin analogues may produce more evident anti-proliferative effects than that achieved by short-term intermittent exposure.

The prediction of shrinkage effects of somatostatin analogues is still controversial. Another factor thought to influence tumor shrinkage is baseline tumor size. Although the literature on this issue is controversial [11], [12], [24] our meta-analysis has revealed that shrinkage of microadenomas and macroadenomas is comparable with octreotide. Nevertheless, the clinical relevance of tumor shrinkage may be greater in macroadenomas compared with microadenomas, particularly considering the excellent results obtained by experienced neurosurgeons with microadenomas [62], [64]. This meta-analysis demonstrated that the shrinkage effect of octreotide correlated with duration of therapy, although the literature indicates that shrinkage may occur in a number of patients after short-term treatment with this drug [34], [44]. This finding may be important when octreotide therapy is proposed for patients with macroadenomas and risk of compression of vital structures.

Biochemical response has also been investigated as a determinant of tumor shrinkage, but data on this question are also controversial [7], [8]. This meta-analysis showed that the prevalence of tumor shrinkage was higher in patients achieving either “safe“ GH levels, as defined by random values generally below 2.0–2.5 ng/ml [57], or normalization of IGF-I. However, our analysis showed that tumor shrinkage may occur even in patients who do not achieve complete biochemical control under octreotide treatment. Indeed, the possible dissociation between tumor shrinkage and biochemical control has been described [9] and may suggest different mechanisms underlying antimitotic and antisecretory actions of somatostatin analogues [56], [65]. In fact, it has been demonstrated that the post-receptor pathways mediating the antiproliferative effects of somatostatin analogues usually differ from those involved in the antisecretory effects of these drugs [1]. Although somatostatin inhibits cell proliferation and may induce tumor cell apoptosis, the mechanisms underlying the direct antimitotic actions of octreotide have not been convincingly demonstrated. Indirect effects of somatostatin analogues on growth factor production and angiogenesis may also be involved [7]. Results of this meta-analysis support the anti-tumor effects of octreotide, a molecule that has also been extensively investigated for treatment of neuroendocrine tumors in different organs [66]. In the future, clarification of the role of different somatostatin receptor subtypes in mediating antimitotic effects [7], may provide a helpful perspective on the effects of the multireceptor-targeted somatostatin analogue pasireotide [67].

A major limitation of our meta-analysis, as for other similar reports [5], [68], [69], was that most trials included in the analysis were open-label and had no control group. Moreover, only few studies aimed at assessing shrinkage as the main endpoint of the study and no specific statistical hypothesis was formulated in many papers. On the other hand, publication bias was not expected and indeed was also excluded by a formal statistical test. Despite these limitations, our results provide a comprehensive perspective on the effect of first-line octreotide therapy on the shrinkage of GH-secreting adenomas in patients with acromegaly. Indeed, clinically significant tumor shrinkage was observed in more than 50% of patients with acromegaly treated with octreotide.

## Supporting Information

Figure S1 **Forest plot depicting the proportion of patients with and without a reduction in tumor size in studies in which tumor shrinkage was evaluated by MRI.** CI, confidence interval.
(TIF)

Figure S2 **Forest plot depicting the proportion of patients with and without a reduction in tumor size in studies with follow-up longer than 3 months.** CI, confidence interval.
(TIF)
