# Supplementary figures and images for: Meta-Analysis on the Effects of Octreotide on Tumor Mass in Acromegaly
Source: PLoS One. 2012 May 4;7(5):e36411. doi: 10.1371/journal.pone.0036411 (PMC3344864; doi:10.1371/journal.pone.0036411)

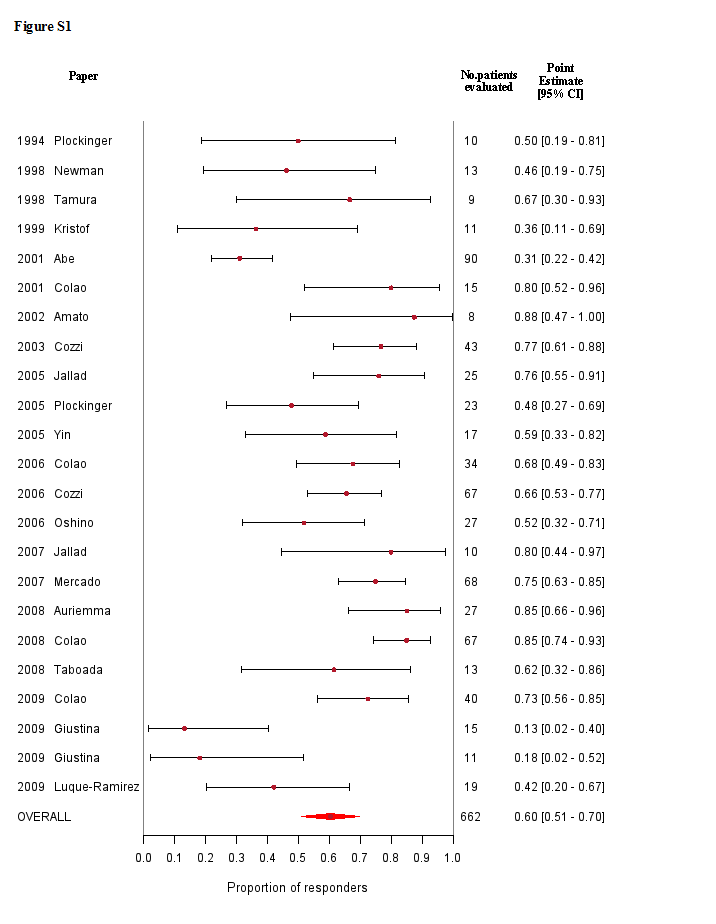

Supplement: Figure S1 — Forest plot depicting the proportion of patients with and without a reduction in tumor size in studies in which tumor shrinkage was evaluated by MRI. CI, confidence interval. (TIF) [file pone.0036411.s001.tif]

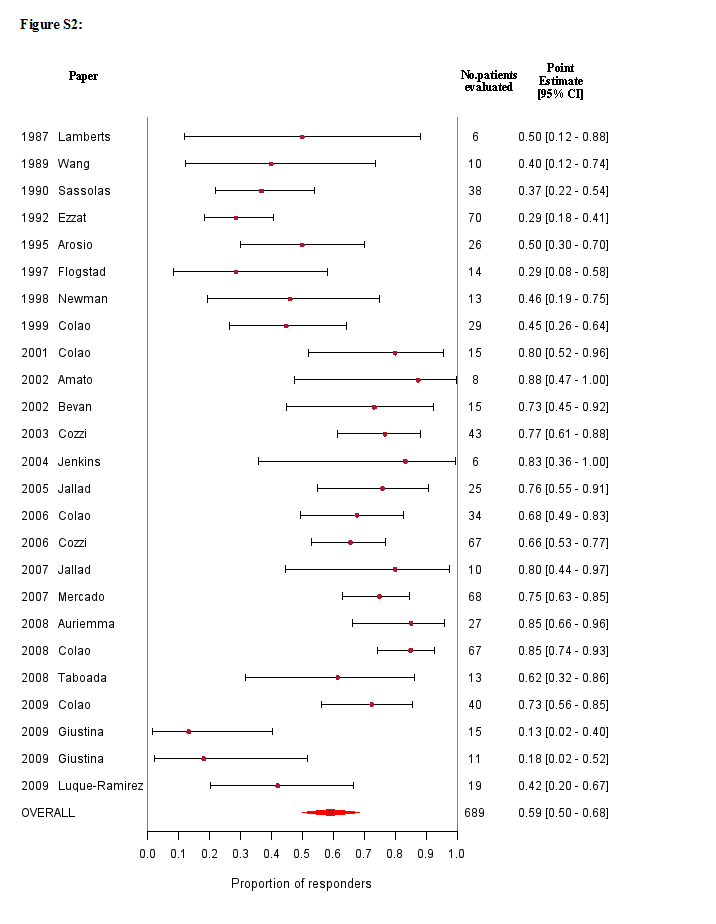

Supplement: Figure S2 — Forest plot depicting the proportion of patients with and without a reduction in tumor size in studies with follow-up longer than 3 months. CI, confidence interval. (TIF) [file pone.0036411.s002.tif]
